# Correction: Life and death of a leprosy sufferer from the 8^th^-century-CE cemetery of Kiskundorozsma–Kettőshatár I (Duna-Tisza Interfluve, Hungary)—Biological and social consequences of having Hansen’s disease in a late Avar Age population from Hungary

**DOI:** 10.1371/journal.pone.0269048

**Published:** 2022-05-23

**Authors:** Olga Spekker, Balázs Tihanyi, Luca Kis, Csaba Szalontai, Tivadar Vida, György Pálfi, Antónia Marcsik, Erika Molnár

The images in Fig 7 are low resolution. The authors have provided a corrected version here.

**Fig 7:**
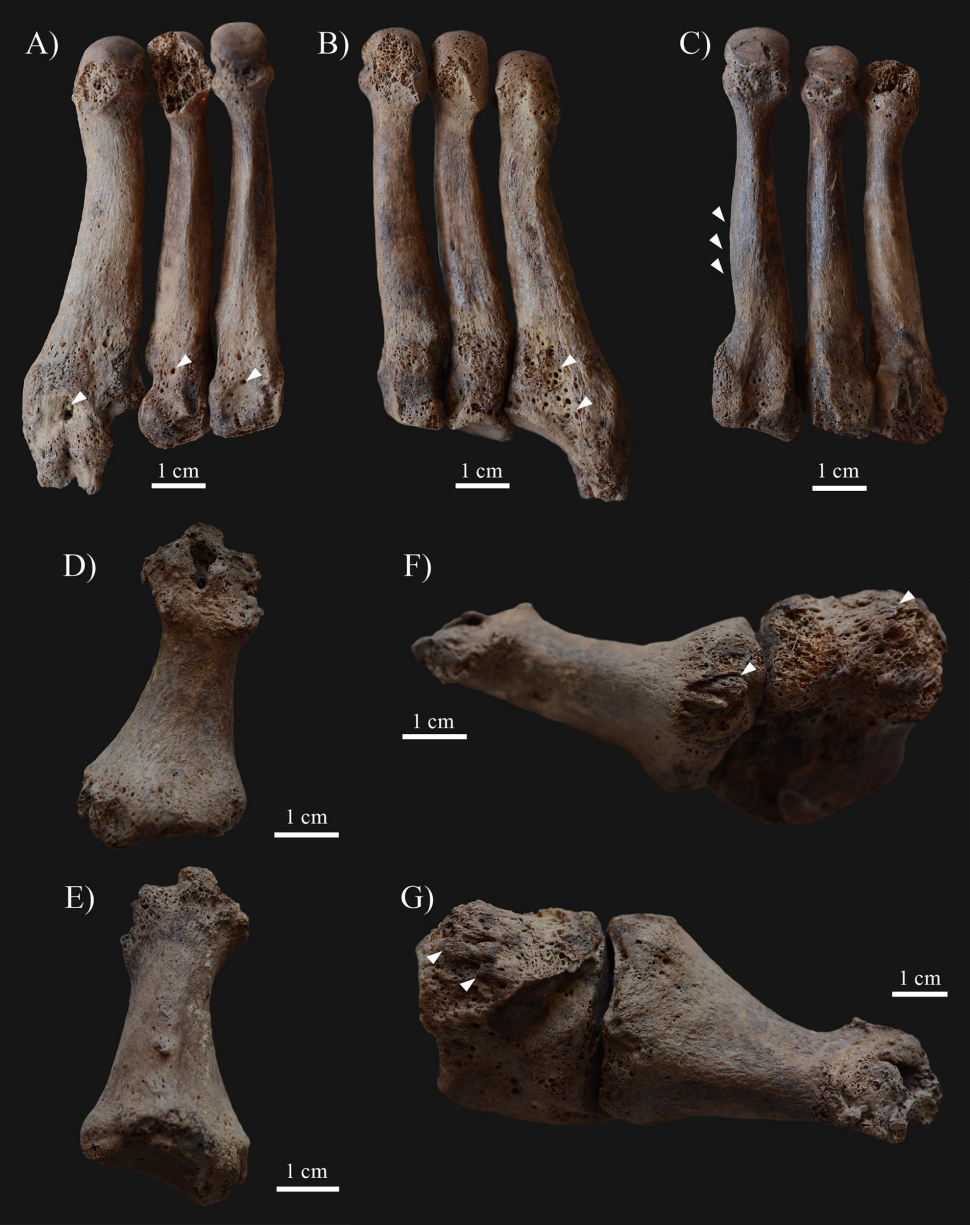
Septic bony changes indicative of sensory peripheral neuropathy in the feet of KK61. Surface pitting and subperiosteal new bone formations on the A) dorsal and B) plantar surfaces of the proximal end of the left 3^rd^, 4^th^, and 5^th^ metatarsals (white arrows), with a small sinus on the 5^th^ metatarsal; C) Slight ballooning of the diaphysis of the right 2^nd^ metatarsal (white arrows); Almost complete destruction of the distal end of the right 1^st^ metatarsal–D) plantar and E) dorsal surfaces; and Remodeling of the proximal end of the right 1^st^ metatarsal and the right medial cuneiform bone with surface pitting and subperiosteal new bone formations (white arrows)–F) right lateral and G) left lateral view.
