# Supplementary material for: Life and death of a leprosy sufferer from the 8th-century-CE cemetery of Kiskundorozsma–Kettőshatár I (Duna-Tisza Interfluve, Hungary)—Biological and social consequences of having Hansen’s disease in a late Avar Age population from Hungary
Source: PLoS One. 2022 Feb 18;17(2):e0264286. doi: 10.1371/journal.pone.0264286 (PMC8856564; doi:10.1371/journal.pone.0264286)
Supplement: S1 Table — (PDF) [file pone.0264286.s001.pdf]

S1 Table: Dental status of KK61, and location, direction, and grade of caries and calculus on his affected teeth.

Upper teeth		Extant or not	Caries			Calculus			Abscess
			Location	Direction	Grade	Location	Direction	Grade	
Right side	M3	+	DEJ	B, M	1	Ø	Ø	Ø	+
			Crown	M	2				
	M2	LP	–	–	–	–	–	–	–
	M1	LP	–	–	–	–	–	–	+
	PM2	+	DEJ	B, D	1	Ø	Ø	Ø	Ø
			Crown	D	1				
	PM1	LP	–	–	–	–	–	–	–
	C	LP	–	–	–	–	–	–	–
	I2	LP	–	–	–	–	–	–	–
	I1	LP	–	–	–	–	–	–	–
Left side	I1	LP	–	–	–	–	–	–	–
	I2	LP	–	–	–	–	–	–	–
	C	LP	–	–	–	–	–	–	–
	PM1	LP	–	–	–	–	–	–	–
	PM2	+	Ø	Ø	Ø	Crown	D	1	Ø
	M1	+	Crown	M	1	DEJ	B	1	Ø
						Crown	B	1	
	M2	+	DEJ	B, D	1	Crown	B, M	1	Ø
	M3	+	DEJ	M	1	Ø	Ø	Ø	Ø
Lower teeth		Extant or not	Caries			Calculus			Abscess
			Location	Direction	Grade	Location	Direction	Grade	
Right side	M3	+	DEJ	B	1	Crown	B, D	1	Ø
	M2	+	Ø	Ø	Ø	DEJ	M	1	Ø
	M1	+	DEJ	D	1	Root	B	1	Ø
	PM2	LP	–	–	–	–	–	–	–
	PM1	LP	–	–	–	–	–	–	–
	C	LP	–	–	–	–	–	–	–
	I2	LP	–	–	–	–	–	–	–
	I1	LA	–	–	–	–	–	–	–
Left side	I1	LA	–	–	–	–	–	–	–
	I2	LP	–	–	–	–	–	–	–
	C	+	Ø	Ø	Ø	DEJ	La	1	Ø
						Crown	all	1	
	PM1	+	Ø	Ø	Ø	DEJ	M, D	1	Ø
						Root	M	1	
	PM2	LP	–	–	–	–	–	–	–
	M1	+	DEJ	D	1	Root	B	1	Ø
	M2	+	Ø	Ø	Ø	DEJ	M	1	Ø
						Crown	Li	1	
						Root	Li	1	
	M3	LP	–	–	–	–	–	–	–

I1 = central incisor; I2 = lateral incisor; C = canine; PM1 = 1st premolar; PM2 = 2nd premolar; M1 = 1st molar; M2 = 2nd molar; M3 = 3rd molar; + = present; LP = lost *post-mortem*; LA = lost *ante-mortem*; DEJ = dentino-enamel junction; B = buccal; M = mesial; D = distal; La = labial; Li = lingual; Ø = not present

Grades of caries: 1 = incipient; 2 = moderate

Grades of calculus: 1 = slight
